# The adolescent experience of hereditary angioedema: a qualitative study of disease burden and treatment experience

**DOI:** 10.1186/s13023-025-03539-0

**Published:** 2025-01-10

**Authors:** Lynne Broderick, April Foster, Laura Tesler Waldman, Laura Bordone, Aaron Yarlas

**Affiliations:** 1 QualityMetric, an IQVIA business, 1301 Atwood Avenue, Suite 216E, Johnston, RI 02919 USA; 2 Ionis Pharmaceuticals, Inc, 2855 Gazelle Court, Carlsbad, CA 92010 USA

**Keywords:** Hereditary angioedema, Disease burden, Qualitative research, Quality of life, HRQoL, Treatment experience

## Abstract

**Background:**

Hereditary angioedema (HAE) is a rare, autosomal dominant disorder causing swelling attacks in various parts of the body, resulting in impacts on health-related quality of life (HRQoL). The symptoms of HAE and its impacts on HRQoL have been well-documented in adults; however, relatively little is known about the experiences of adolescents with HAE. The objective of this study was to use qualitative interviews to investigate how adolescents experience HAE symptoms and how HAE impacts their HRQoL.

**Methods:**

This was a non-interventional, qualitative study of adolescents with HAE. Participants were recruited via a patient advocacy organization and were eligible to take part in this study if they had a confirmed diagnosis of type I or type II HAE and were currently on prophylactic treatment to prevent HAE attacks. All participants completed a one-to-one, 60-minute, remote interview designed to elicit their experiences of HAE. Interview data were coded and analyzed using NVivo qualitative software.

**Results:**

Twelve adolescents took part in this study. HAE attacks were described as painful and uncomfortable. Attacks varied by trigger, frequency, severity, location, and duration. Participants described ways in which HAE impacted their daily lives, including impacts on physical, social, emotional, and cognitive functioning, in addition to sleep disturbance, school-related impacts, and a need to avoid attack triggers. Impacts on emotional and social functioning were particularly noteworthy, as participants reported having to miss or skip social events, and sometimes withdrawing socially. Since initiating prophylaxis, participants reported the frequency, severity, and duration of attacks had been reduced and their HAE-related impacts had been minimized. Participants were satisfied with their current prophylactic and acute treatments, and expressed a preference for treatments that were effective, convenient, self-administered, and had minimal side effects.

**Conclusion:**

Adolescents with HAE reported experiencing a range of symptoms that, when untreated, impacted their HRQoL in ways that are unique from adults. Further, participants reported that effective treatments (prophylactic and acute) inhibited symptoms and HRQoL impacts with minimal treatment burden. Findings from this study suggest that health care providers and clinical investigators should consider the unique HRQoL impacts experienced by adolescents when evaluating treatment benefit.

## Background

Hereditary angioedema (HAE), a subtype of bradykinin-mediated angioedema (AE), is a rare, autosomal dominant disorder. Type I HAE, which accounts for the majority of cases, is associated with C1 esterase inhibitor protein deficiency; type II results from a dysfunctional C1 inhibitor protein [1, 2]. An estimated 1 in 50,000 to 1 in 100,000 individuals worldwide and 1 in 10,000 individuals in the United States (U.S.) have HAE [3, 4]. Recent studies have reported the mean age of HAE symptom onset to be between 10 and 12 years old, with a range of 0–58 years old [5, 6]. 

Clinical HAE characteristics include recurrent subcutaneous and submucosal swelling episodes, or attacks, most often in the extremities, abdomen, genitourinary tract, face, and larynx [7]. Attacks can range from mild to severe in intensity, with laryngeal episodes being potentially life-threatening due to the risk of asphyxiation [8]. Though HAE attacks can be unpredictable, common attack triggers include hormone changes (e.g., puberty), stress, fatigue, and physical trauma [7]. 

The impact of HAE on patient health-related quality of life (HRQoL) is largely dependent on the frequency and severity of attacks. While experiencing HAE attacks, adults frequently report pain and disfigurement, which can lead to restrictions in everyday activities [1, 8–12]. Further, adults with HAE can experience disruptions with social interactions and reduced productivity as a result of absenteeism and presenteeism [1, 6, 8, 11, 13]. In between attacks, adults with HAE may experience emotional distress, including anxiety due to the unpredictability of attacks and potential for fatal laryngeal attacks [1, 6, 8, 11, 13], and concerns surrounding their children inheriting HAE [8, 11]. Though HAE attacks can be debilitating, prophylactic treatments have been shown to decrease their frequency, improve HRQoL, and reduce disease burden [9, 10, 12]. 

While the substantial burden and impact of HAE on adults’ HRQoL are well-documented [1, 6, 8–12, 14, 15], relatively little is known about the experiences of adolescents with HAE. Recent survey studies have investigated HRQoL of children and adolescents with HAE as compared to healthy controls; [16, 17] however, there is a lack of qualitative studies focused on their unique disease and treatment experiences. Qualitative studies provide the opportunity to directly capture the patient’s voice and explore their experiences in-depth, including identifying the aspects that are most meaningful to them, and that have not yet been thoroughly researched [18]. In its Patient-Focused Drug Development Guidance for Industry, the U.S. Food and Drug Administration underscores the importance of using qualitative studies to fully understand the patient experience [19]. Using a qualitative study in this way will more clearly characterize the adolescent burden of disease and potential unmet treatment needs.

### Objective

The objective of this study was to use one-to-one, qualitative, concept elicitation interviews to investigate the ways in which adolescents experience HAE symptoms and how HAE impacts their HRQoL.

## Methods

This was a non-interventional, qualitative study that involved adolescents identified via a US-based, HAE-specific, patient advocacy group (PAG). The study, including a waiver of documented consent (parental consent and minor assent), was approved by WCG IRB.

### Recruitment of patients with HAE

The PAG invited patients to take part in this study by email. Eligible participants had to be between 12 and 17 years old, have been told by a physician they have type I or type II HAE, be taking prophylactic HAE treatment at the time of the study^1^, live in the U.S., speak and read English, and provided confirmation of their HAE diagnosis. Patients were excluded if they were diagnosed with HAE with normal C1-inhibitor. The recruitment goal was to enroll equal numbers of adolescents by age group (12–14 years old and 15–17 years old) and by self-identified sex at birth (male/female/other).

To protect the privacy of its members, the PAG did not share any identifiable patient information with the research team. The PAG assigned each enrolled individual a study identification number and a pseudonym to use during the interview. Participants’ names and any other identifying information were stored securely by the PAG and kept separate from any data collected.

### Interviews

Interviews were conducted one-to-one remotely with study participants by 1 of 3 qualified members of the research team; all interviewers were trained on the study specific interview guide and participated in mock interviews prior to conducting any participant interviews. All interviews were about 60 min in length and conducted using web-conferencing software, which allowed participants the options of joining by phone, tablet, or computer, with or without the use of a webcam. All interviews were audio-recorded with permission and transcribed verbatim by a third-party vendor. Once each interview was complete, the PAG was notified and subsequently disbursed an honorarium ($100) directly to each participant.

Because all participants were under the age of 18, a parent was asked to be present for the beginning of each interview so the interviewer could review the study details and obtain verbal parental consent before obtaining verbal assent from the adolescent participants. At their discretion, parents could then choose to remain present during their child’s interview. Interviewers informed any parents who opted to remain present that hearing directly from their child was of utmost importance to the study and asked them to not answer any questions on behalf of their child.

Each interview followed a semi-structured guide that was developed specifically for this study using input from clinical experts and reviewed by the PAG prior to data collection. The guide was designed to capture participants’ experiences with HAE in their own words, and included a series of questions intended to elicit descriptions of HAE symptoms, impacts of HAE attacks on HRQoL, and treatment experiences related to the use of prophylactic and acute treatments. Interviewers used their discretion to probe further into different aspects of participants’ responses for elaboration and clarification.

### Data analysis

Demographic and clinical characteristics of the study sample were summarized in a table and presented using counts and percentages for categorical variables.

Interview data were coded and analyzed in NVivo qualitive software (v.12; QSR International Pty Ltd., 2018). Coding is the process by which researchers reviewed transcripts to identify and organize the concepts that emerged during the interviews. The research team developed the coding structure using the first 2 transcripts. Following agreement of the coding structure, the team met regularly throughout coding to address any questions regarding coding or the coding structure and to maintain agreement throughout the process.

Inductive thematic analysis [20], in which specific themes emerge from the data, was used to identify patterns in participant responses concerning relevant and important elements of their HAE experiences. Participants’ accounts were summarized according to the concepts that emerged from the interviews. These summaries were then used to characterize the adolescent experience of HAE, specifically including the symptom experience, the impacts on feeling and functioning, and the treatment experience.

### Saturation

Saturation, a process used to assess the emergence of new concepts across consecutive interviews, is reached when additional interviews no longer contribute new concepts or meaningful information about the shared patient experience [18]. To assess saturation, interview transcripts were placed into 4 groups of 3 ordered chronologically by interview date. The assessment of saturation showed that 90% of concepts emerged by the 9th interview, indicating that 12 interviews provided sufficient data to inform the understanding of the concepts being explored, and that saturation had been reached.

## Results

### Patient characteristics

Twelve adolescents with HAE took part in this study (Table 1). The study sample was split evenly by age (*n* = 6 adolescents aged 12–14; *n* = 6 aged 15–17; 50.0% each) and sex (*n* = 6 male; *n* = 6 female; 50.0% each). Most participants were non-Hispanic white (*n* = 11; 91.7%).

**Table 1:** Patient characteristics (*N* = 12)

	*n* (%)
**Age/Sex**	
**12–14 Years**	6 (50.0%)
Female	3 (25.0%)
Male	3 (25.0%)
**15–17 Years**	6 (50.0%)
Female	3 (25.0%)
Male	3 (25.0%)
**Race/Ethnicity**	
Non-Hispanic White	11 (91.7%)
Hispanic/Latino	1 (8.3%)
Treatment*	
Prophylactic	12 (100%)
Acute/On-demand	11 (91.7%)

*One participant reported not taking an acute treatment and 2 reported taking 2 acute treatments

### Adolescent experience of HAE attacks

#### Symptoms and locations of attacks

HAE attacks were described as painful and uncomfortable. Participants noted that their experiences could vary from one attack to another. As one 12-year-old female described, “sometimes it’s painful, sometimes it’s itchy, sometimes it’s just swelling” (see Table 2). In addition to swelling, participants frequently reported experiencing pain (*n* = 10; 83.3%), nausea (*n* = 7; 58.3%), and vomiting (related to abdominal attacks; *n* = 5; 41.7%) during attacks. A second 12-year-old female described abdominal attacks as, “…my stomach—it would just like—feel like, um—squeezing, like it would hurt a lot and I would be vomiting.” Additionally, participants reported rash (*n* = 5; 41.7%), fatigue (*n* = 4; 33.3%), dizziness (*n* = 3; 25.0%), heartburn (*n* = 2; 16.7%), itch (*n* = 2; 16.7%), a “tingling sensation” (*n* = 2; 16.6%), vomiting (unrelated to abdominal attacks; *n* = 1; 8.3%), loss of appetite (*n* = 1; 8.3%), and fever/hot flashes (*n* = 1; 8.3%).

**Table 2:** Quotes exemplifying the adolescent experience of HAE

Symptoms and Locations of HAE Attacks	I always would get [an] attack in my stomach. So, I would um—vomit a lot…I have been told that I like passed out while like puking…[I] would start get a pain…at the bottom of [my] ribs…like in between them, in like that area. [I] would start to get like a stomachache…[my] stomach would get swollen and then…got like dizzy a bit and then like vomit…Oh, and um—splotches…whenever [I] would get a stomachache, [I] would check for splotches, kind of like reddish rings that would for some reason went all over [my] back. (male, age 14) …my hands are just getting tight, like tighter and like uncomfortable…if they were to be left untreated…they’d get as swollen as they can, but they’re still like well sausage fingers (female, age 17) …my whole face just swells. I—I can’t recognize myself in the mirror really, it’s that—it gets that bad…It’s very painful…my like eyelid—I usually can’t see out of my eye usually because it’s swelled so bad…it usually goes to my lips and then it’s just—it’s—it’s painful…when I had it in my face, it was bad. I mean it—I looked in the mirror and I, I couldn’t tell I was me. I couldn’t tell for 4 days that, that was me. (male, age 16) I had one in my throat, er, a few—maybe 4 years ago…it was scary because um—like I was just sitting in my basement and then my throat started like feeling—it—I can’t really describe the feeling, but it just felt like weird…and then it started feeling like sore a little bit. And then um—like I started to feel like I wasn’t really able to, to breathe like normally…And then like it started to become like harder for me to breathe but I was still able to breathe and talk, um—and then um—I told my mom, and we did my medicine instantly because we didn’t want to risk anything…and then we drove to the hospital…. (male, age 17)
Most Bothersome Symptoms	The stomach one…they hurt the most. I’m not able to like fully function with them. Like I can move around like a little bit but like if it’s just in my hand or foot, it’s a little uncomfortable to like do normal like daily tasks, but if it’s in the stomach, I pretty much can’t do anything. (male, age 17)
Attack Triggers	…trampolines, like if I’m jumping for too long, my stomach used to—my stomach would swell…if I’m walking too long, like my feet could swell…crutches—I couldn’t use that…my hands and my arms would swell from the crutches…my inner thighs would used to swell when I would go on um—like bikes…I had to get like a special bike seat, ‘cause my thighs would swell. (female, age 12) I still do play a lot of [video] games and I would play with this controller, and every so often, my thumb would get all like stiff, because if you play with controller, your thumb and fingers get stiff sometimes. Most of the time, if I wasn’t on medication, my thumb would start to swell and then it would slowly turn into my hand. (male, age 13) I’ll occasionally have like a hand or a foot swell when the weather’s like really humid…I do an outdoor er—sport and like in the mornings if we’re having practice, my fingers will get all puffy because like I don’t really know, but [laughs] I just—and like if I’m walking outside and it’s humid, my hands will swell up. Like it’s—it’s probably activity outside when it’s humid. (female, age 17)

Abbreviation: HAE, hereditary angioedema

**Fig. 1:**
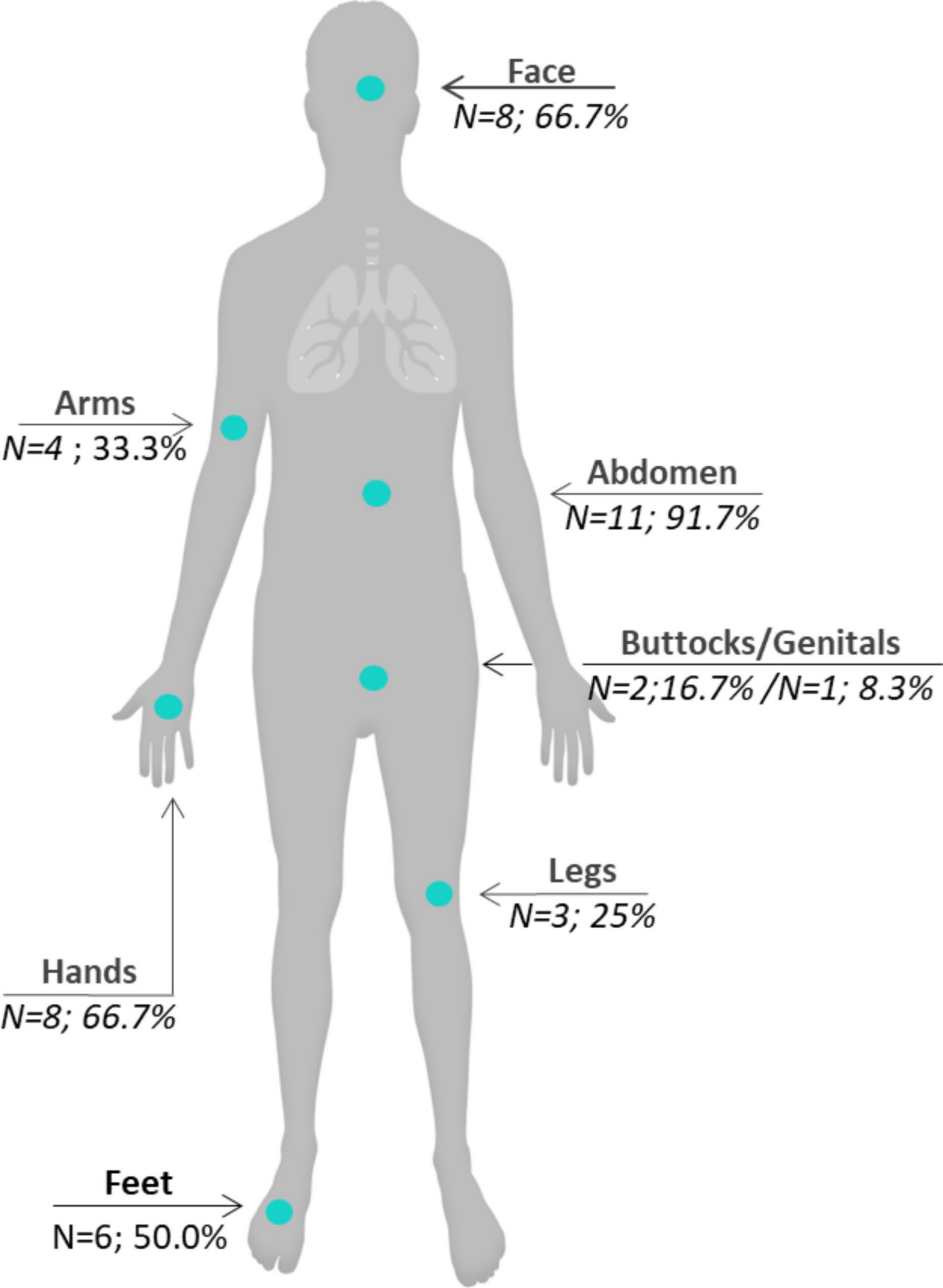
Bodily locations of HAE attacks Abbreviation: HAE, hereditary angioedema

Participants experienced attacks in various bodily locations (Fig. 1), with nearly all reporting abdominal attacks (*n* = 11; 91.7%).

#### Frequency and severity of attacks

Participants described the frequency of their attacks in terms of before versus after starting their prophylactic medication regimen. Pre-prophylaxis, participants reported a wide variety in attack frequency, ranging from weekly to monthly to a few times per year. Post-prophylaxis, the frequency decreased dramatically, and attacks were rare. As one 17-year-old female noted, “I don’t have them very like often at all because I am well-treated and controlled right now…I’ll occasionally have like a hand or a foot swell when the weather’s like really humid…Before, I was having stomach swells, like 2 or 3 times a week.”

The severity of attacks varied greatly depending on the location and participant. Participants described attacks in the abdomen, face, and larynx as more severe; abdominal attacks were also reported to be incapacitating, while laryngeal attacks were described as scary. Attacks in the hands, feet, arms, and legs were generally perceived as less severe and/or more manageable, but that perception depended on the level of functional impairment (e.g., ability to use hands/fingers) experienced with the attacks.

#### Most bothersome symptoms

When asked about their most bothersome symptom, of which they could report more than one, participants most frequently reported the pain associated with any swelling (*n* = 7; 58.3%): “I would say the pain…that’s honestly the only thing that’s really limiting me from doing—like going through my regular like everyday life.” (female, age 17). Abdominal swells (*n* = 5; 41.7%) and vomiting/nausea associated with an attack (*n* = 2; 16.7%) were also noted to be most bothersome symptoms. Similarly, participants identified pain (*n* = 4; 33.3%), abdominal swelling (*n* = 2; 16.7%), swelling (in general; *n* = 2; 16.7%), and vomiting (*n* = 2; 16.7%) as the symptoms most important for their treatment to prevent or relieve.

#### Attack triggers

Attacks were triggered most commonly by stress (*n* = 9; 75.0%). Participants also reported HAE attacks being triggered by injury (*n* = 5; 41.7%), prolonged or repetitive activity (e.g., jumping on a trampoline triggered an abdominal attack, playing video games triggered an attack in the hands; *n* = 4; 33.3%), heat or becoming overheated (*n* = 4; 33.3%), being sick (*n* = 2; 16.7%), and hormonal fluctuations (*n* = 1; 8.3%).

### Impacts of attacks on HRQoL

**Fig. 2:**
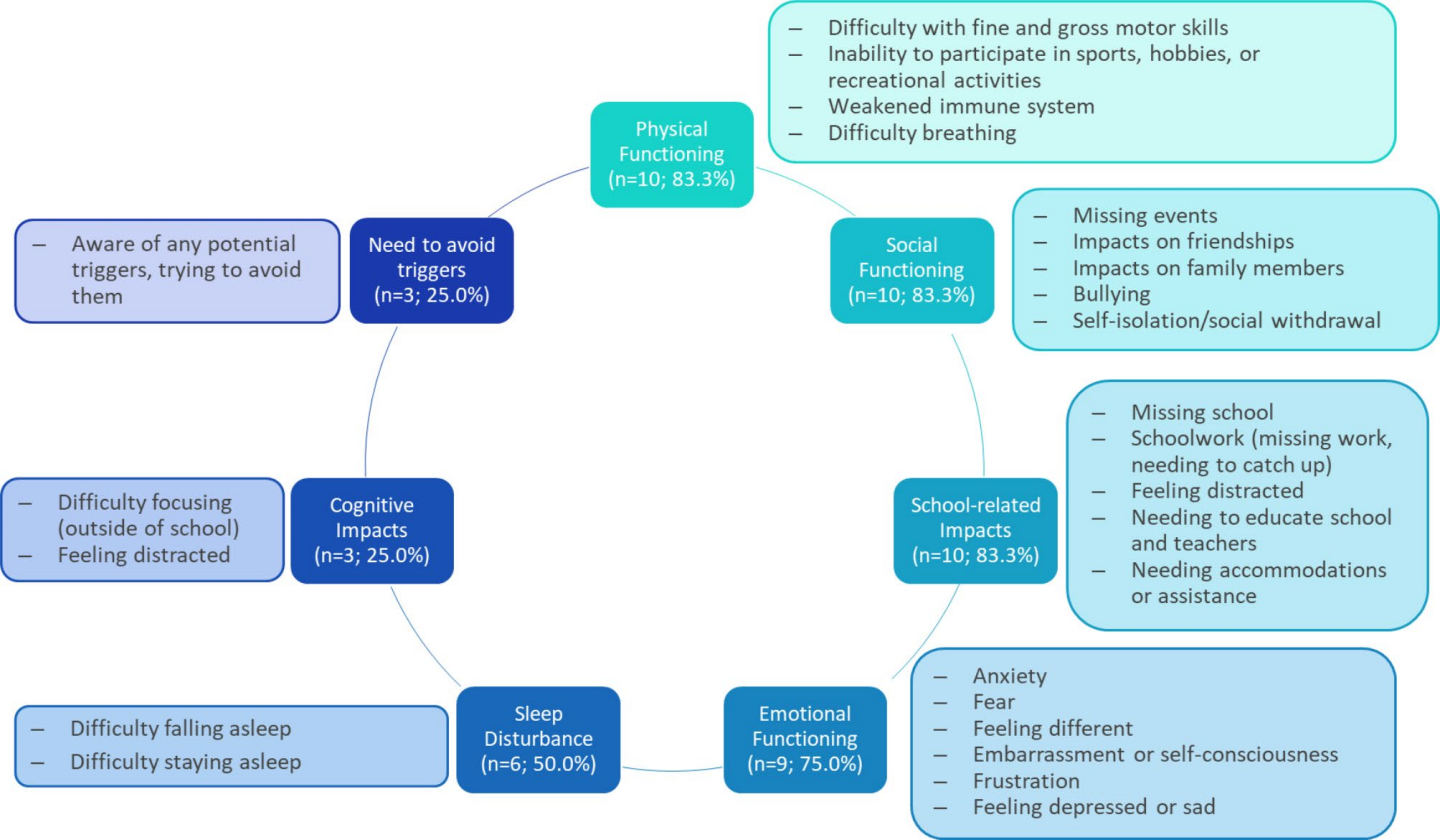
HRQoL domains impacted by HAE Abbreviation: HAE, hereditary angioedema

Participants reported experiencing HAE attack-related impacts across 7 domains of HRQoL (Fig. 2). Similar to symptoms, HRQoL impacts were mitigated by prophylaxis and described as less burdensome since beginning treatment. One 12-year-old female participant described, “[I] could never really do sports because [my parents] were nervous that I was gonna get …injured in some way… I couldn’t really do sports until I got preventative medication.” A second participant (17-year-old female) shared that their prophylactic treatment reduced the interference of HAE in their daily life such that, “[I] forget that I have HAE, and like it’s not a thought in the front of my mind every single day.”

Physical functioning impacts were described as interference with fine and gross motor skills (e.g., difficulty writing or typing/using a phone due to hand swelling; difficulty walking due to foot/leg swelling; see Table 3 for example quotes). Participants were unable to try out for or fully participate in sports (i.e., missing practices and/or games). As described by a 17-year-old male, “[HAE] definitely hindered like my ability to ever think about trying out for sports. Um—I’m just too nervous to for like the overall risk because like, sometimes like—most of the time actually, physical activity does sometimes make it worse.” Hobbies (e.g., riding bikes) and some recreational activities (e.g., going to the beach, attending sleepaway camp) were also limited due to the threat of attacks. In addition, participants reported feeling short of breath, which made everyday activities like climbing stairs challenging; they also observed that they were often sicker than their friends, finding that it took them longer to recover from colds or other illnesses not related to HAE.

**Table 3:** Quotes exemplifying HRQoL impacts of HAE

Physical Functioning	…we used to go to church, we—they had a few activities on hot summer days that I was able to go to but couldn’t stay very long. Probably only about 30 min of the activity I got to stay for because it got too hot—I got too hot and then started to swell. (female, age 12) …definitely hindered like my ability to ever think about trying out for sports. Um—I’m just too nervous to for like the overall risk because like, sometimes like—most of the time actually, physical activity does sometimes make it worse. (male, age 17) if my hand swells, it’s puff—like big and I can’t move it, and I can’t—and it hurts…it hurts to put press-pressure, um, so I can’t really use my hand…writing, um—when I—like this hand was really swollen and I would have to write with like my left hand and I got—I got really—I got good at writing with my left hand… and typing, like I can use one hand to type. Or someone would write for me, at home, or the [school aide] would help me. (female, age 12)
Social Functioning	I’ve definitely missed out on things, like the time that I had the um—laryngeal attack…I was supposed to—there was this big like—like I don’t know, like 30 girl sleepover at this girl’s house that I was invited to and…it was the day after I had the um—laryngeal attack and for some reason I was like—thought it was a good idea for me to um—sleep—still go to the sleepover and my parents were like “No way, you just had like a throat swell, like you could still er, like—” I don’t know, there are things like rebound attacks and stuff and they just wanted to watch me and stuff because there’s—especially ‘cause it was the first time I’d ever had one. So, they didn’t know like what to expect. And I was just—I was really upset because it was like a fun, social thing that I just completely missed out on (female, age 17) I used to get made fun of too whenever like I would swell in my face… I would get made fun of for like you know, coming to school with like you know, a swell on my face and since it’s not really well known, people don’t really know about it, so there’s a lot of like stigma around stuff like that. (male, age 17)
School-related Impacts	…when my attacks were—were pretty severe, like in middle school, um—I think it was either 7th or 8th grade year, I missed about 40 days of school… I would say that catching up on work um—was pretty difficult. Like especially if I’m missing days at a time. (male, age 17) …if I had a feeling that an attack was coming… like I wouldn’t go to school because I know if I went to school, like my—my nurses would have no idea what to do. And so, like I wouldn’t go to school. (female, age 16) I would just overthink it, like the entire elementary—entire elementary school, after 2nd or 3rd—after 2nd Grade, all I used to think about HAE ‘cause I don’t wanna swell, I didn’t—that’s all I’d think about over and over again and then I would get bad grades and stuff—like just reading, um—books and stuff, I would just start dozing off and start thinking about HAE (male, age 13)
Emotional Functioning	I’m just hoping like it never progresses worse, but they said it could. And it’s just like a wait and see thing. So, I guess like the uncertainty of it, and just the waiting is always just kind of been a little scary. (male, age 17) Sleepovers, growing up, I would—it kind of sucked for me because I would always like get like anxiety in a way … I would sometimes go home early from sleepovers or not even have sleepovers (male, age 13) …with that friend that stopped our friendship due to a swell, it in the moment made me feel like my disease was my fault and it’s really not a big deal…even I do have a horrible swell, I can do—I can still do stuff and it did kinda make me feel abnormal. (female, age 12)
Sleep Disturbance	it definitely disrupts my sleep a lot. I wake up like several times throughout the night and like I can just feel it like in my face, like—I like get up and I’ll look in the mirror and it’ll just be there. (male, age 17) …if the attacks come at night, I won’t sleep because then I get scared like ‘cause like for some people, like their throat will swell up and I don’t know if one day that will happen to me. So, I don’t like to go to sleep if I’m having an attack. (female, age 16)
Cognitive Impacts	I couldn’t really focus on anything. Like maybe I was like 6 or 7, I wanted to watch like a YouTube video to like distract myself, I would have to like—after maybe—after a while, I would find that that I would get focused on that but I would usually still be hurting in my stomach for a while (male, age 14)

Abbreviations: HAE, hereditary angioedema; HRQoL, health-related quality of life

Social functioning was primarily impacted by participants having to miss or skip events, including spending time with friends, sleepovers, birthday parties, school events, and family events. One 16-year-old female shared:

I was…with my friends. I just had to go home…I knew it was coming just because…I could feel… my stomach starting to hurt a little bit and I [knew I needed] to go home. So, it stops me from like—if I know something’s coming…I can’t even continue what I’m doing.

Attacks caused participants to withdraw socially, beyond having to miss events; they reported wanting to “stay in their room” and not see anyone. While some participants discussed sharing information about HAE with their friends, including teaching friends how to inject their medicine, others reported losing friends or being teased because of their swelling episodes. One 13-year-old male participant recalled an experience in which an older child repeatedly threw a ball at him, ostensibly with the intent of triggering an attack:

…he started doing this thing ‘cause my mom would like talk—like tell people like don’t even like you know, playfight or anything, just ‘cause I don’t wanna get a swell… I was hiding in the slide, ‘cause I didn’t wanna get hit by the ball, and he was yelling at me… and he kept throwing this ball, trying to hit me.

Some participants described impacts on family members, feeling their HAE caused stress and worry, or that attacks changed family plans such that everyone had to miss an event or stay home from work to care for them. As one 17-year-old female explained, “…it sometimes messes up my family’s life because I don’t know, usually they wanna have somebody at home with me and sometimes like people have to like take off work or like cancel things to stay home with me.”

Participants also experienced school-related impacts, including attending school, keeping up with schoolwork, and being able to focus and concentrate while at school. In some instances, their school and/or teachers were not supportive of their HAE-related needs (e.g., being reprimanded for removing a shoe during class due to a foot swell); as a result, they had to advocate for themselves and teach school nurses and faculty about HAE. One 17-year-old male described:

…my school hasn’t really been all that understanding with me being diagnosed with HAE, especially because they’ve never heard of it, and we’ve actually been struggling for like a few months, trying to even have my emergency medicine at the school. So, if I have an attack, I have to be checked out of school and go get my medicine from somewhere else. Instead of being able to have it on me right away.

Emotionally, participants reported feeling fear and anxiety about HAE attacks due to their unpredictability in both frequency and severity. A 17-year-old male explained, “I’m just scared like you know, one day my medicine will be too far [away], and I’ll have like a life-threatening attack and like I know it could kill me.” Participants also reported they sometimes just felt “different” from others their age and were sometimes embarrassed or self-conscious about having HAE, particularly if they had an attack that was visible to others. One 13-year-old male noted that for a period of time, he saw a therapist:

…because it made me so sad and I felt like, like there was something wrong—wrong with me…because I would see all—all these kids and stuff at recess, you know, running around, doing their thing, being able to scream and stuff, and I would just be not really trying to do much ‘cause I don’t wanna swell up.

Sleep disturbances were related directly to HAE attacks. Participants had difficulty falling asleep during an attack, not only due to the discomfort, but fear that the attack might worsen. Participants further reported waking up in the middle of the night mid-attack and needing to go to the hospital or be treated at home to stop the attack.

In terms of cognitive impacts, participants reported feeling distracted both in and outside of school, either due to an attack or due to worrying about an attack. Finally, participants described a need to be vigilant about preventing attacks when possible and a need to assess situations to identify any potential triggers. As one 14-year-old male described:

…when I was younger, I would like—I would think like um—can I do this? Like could it trigger an attack or if I swell—swelled, would it be very, very bad? …a year ago I had to like get braces…like could braces cause irritation? Would we have to go with Invisalign? …I didn’t know if like putting these brackets on my teeth could swell and then swell in my mouth and my face and that could be bad. So, I had to worry about like that stuff.

In addition to the previously-described HRQoL impacts, 1 participant shared that they recently discovered they could not join the military due to HAE, impacting their plans for the future. Not all impacts reported were negative, however, as 5 participants noted that HAE positively impacted their lives. These participants reported positive incidents such as teaching their friends about HAE, working in patient advocacy to assist others with this condition, and finding and forming relationships with others their age with HAE. They also noted that they were no longer afraid of needles/injections and have become less susceptible to pain.

### Treatment experiences

Overall, participants reported satisfaction with both current prophylactic and acute treatments, particularly expressing gratitude for prophylactic treatments that enabled them to live a “normal life” (female, age 12) (see Table 4 for additional quotes). Participants noted that since taking prophylaxis, they experienced less frequent attacks, less severe attacks, and less daily interference from HAE. When asked what they liked most about their prophylactic treatments, participants reported the ability to self-administer, which allowed for a sense of independence and control (*n* = 9; 75.0%); efficacy (*n* = 8; 66.7%); and convenience (ease of administration, and, for some, infrequent administration; *n* = 8; 66.7%). Least liked characteristics included side effects (pain or soreness at the injection site, upset stomach; *n* = 8; 66.7%), and inconvenience (time-consuming to administer, need to take it with food, frequent administration; *n* = 4; 33.3%).

During interviews, all participants reported receiving acute treatments for HAE attacks, (11/12 reported using an acute treatment during screening) either in the Emergency Department, at home by a parent, or both. Pain (particularly abdominal), or a sense an attack was worsening, such as spreading to the throat or tongue, were reported as key indicators for taking an acute treatment. Most participants (*n* = 9; 75.0%) reported consulting with a parent about whether they needed treatment. If a participant felt any abdominal pain, acute treatment was often administered, “just in case” (female, age 13). When asked what they liked most about their acute treatments, participants identified efficacy (*n* = 8; 66.7%), ease of administration and storage (*n* = 4; 33.3%), and that it helped them overcome their fear of needles (*n* = 1; 8.3%). Dislikes included inconvenience and administration being time-consuming (*n* = 8; 66.7%), side effects (pain or soreness at injection site, upset stomach; *n* = 4; 33.3%), and unreliable efficacy (*n* = 1; 8.7%).

**Table 4:** Quotes exemplifying the adolescent HAE treatment experience

Prophylactic Treatment	I can live like a normal life with [HAE] and I didn’t—it doesn’t—like it doesn’t have to—my HAE doesn’t have to inter—like interfere with my life anymore and so I wouldn’t have any at—anymore attacks, and so it’s less painful also, like so I don’t get attacks… it’s helping me definitely so I’m grateful for it. (female, age 12) I don’t even think about HAE anymore actually. This is the first time I’m talking about HAE in a long, long time after—after I got [treatment], the new medication it helped me a lot. So, I don’t even think about HAE anymore. I kinda forgot that I even had it. Um—I only get reminded about it once I take my shot every month. (male, age 13) …I guess the like—sensation of it going in. It’s definitely not like pleasant. I guess I’d say that’s my least favorite thing but it’s definitely like I can deal with it. (female, age 17)
Acute Treatment	…it worked very fast. It took like 10 min to work…and then it also doesn’t have to be refrigerated so like you can just carry it in your bag with you. (female, age 17) …it’s just really like easy. It’s not painful at all. Just really easy. (male, age 14) I would say to just like how well it works because it’s—it’s very reliable and it always—it always works and I never get any rebound attacks so I think that’s—that’s the—that’s the reason why I’m still using it. (female, age 17) …how long it takes to do. That one, like to push it and like finish the medication, because like once you do the, um—the IV, you have to like push the saline and then for me, um, I—um, I’m prescribed 3 boxes like per dose, so like we have to push all those vials of the medicine and then do another saline. So that the thing itself usually take about half an hour… Yeah, so the whole thing is about an hour. (male, age 17)

Abbreviation: HAE, hereditary angioedema

## Discussion

The purpose of this study was to use one-to-one, concept elicitation interviews with adolescents with HAE to learn about the symptoms they have, the ways in which HAE impacts their HRQoL, and their experiences with prophylactic and acute treatments for HAE attacks. Although the adult HAE experience has been well-documented, to date it has mostly been assumed that the adolescent experience mirrors that of adults. Findings from the current research suggest that while the experiences of adults and adolescents are similar in many ways, there are also unique aspects of being an adolescent with HAE, particularly the impacts on social and emotional functioning and schooling.

The HAE attacks described by adolescents in this study, including symptoms, bodily locations of attacks, triggers, frequency, and severity, were similar to those of adults as described in the literature [6–8, 21]. Perhaps unique to adolescents, and potentially overlooked by clinicians and the literature when trying to identify causes of HAE attacks, were some of the reported triggers that included physical/recreational activities leading to abdominal and upper thighs/buttock attacks, and playing video games causing hand attacks.

Participants experienced impacts in multiple HRQoL domains, although perhaps most notable were impacts on their social and emotional functioning and impacts related to school. Socially, participants felt different from their peers and reported missing “typical” childhood events such as sleepovers, birthday parties, and spending time with friends. Additionally, HAE attacks resulted in some adolescents proactively withdrawing socially, isolating themselves to avoid potential attack triggers, or avoiding being seen if they were experiencing swelling. Some participants reported feeling self-conscious and experienced episodes of bullying or teasing due to being different or having a visible swell. Although the literature examining the social functioning of adolescents with HAE is lacking, recent studies and review articles have documented similar findings of feeling different, isolated, and self-conscious in children who have rare diseases, including lymphoedema which can also lead to disfiguring swelling [22–24]. 

Emotionally, participants described their fear and anxiety that an HAE attack may happen at any time and explained that it is not possible to predict the location or severity of an attack. This aligns with recent studies examining anxiety and HRQoL in children with HAE [16, 17]. In their study, Kessel, et al. [16] found children with more frequent and severe HAE attacks to have higher anxiety, and that stress was the most common trigger for abdominal attacks. While causality cannot be inferred (i.e., are more frequent/severe attacks causing anxiety or is anxiety causing more frequent/severe attacks), it is important to note the link between HAE attacks and emotional functioning. Similarly, Engle-Yeger [17], et al. found overall HRQoL in children with HAE was related to the number and type of HAE attacks they experienced. Feelings of anxiety and fear that their condition may worsen have also been observed in children with other rare diseases [24]. 

Participants in the current study also described the ways in which HAE attacks impacted their education and school experience, including missed days of school and falling behind, similar to findings reported by Engle-Yeger, et al. who found that impacts on schooling were strongest for children experiencing multisite HAE attacks [17]. Participants in this study further noted a general lack of knowledge and understanding among school faculty and administrators, which has been reported in studies examining HRQoL of children with rare disease [22, 23]. 

When describing their HAE attacks and HRQoL impacts, participants were mindful to state that their experiences have been very different since being on prophylaxis. This aligns with studies of adult populations showing a decrease in HAE-related burden after initiating prophylactic treatment, including fewer attacks and improved HRQoL [9–11], and underscores the importance of data collected specifically on adolescents’ treatment experiences. All adolescents in this study were taking medication to prevent HAE attacks. These treatments enabled them to manage daily life without HAE symptoms and removed several limitations (e.g., inability to take part in sports, social events, school) that had been impacting their HRQoL.

This study was not without limitations. Demographically, the sample was mostly non-Hispanic white, so the findings may not be representative of all adolescents with HAE. Additionally, some participants had been taking prophylaxis for a number of years and reported having experienced severe attacks only when they were much younger, thus there may exist a potential recall bias in their accounts of both symptoms and impacts on HRQoL. Finally, to avoid influencing participants’ perceptions of their HAE symptoms and impacts on HRQoL, they were not asked to complete an objective measure of HAE symptoms or HRQoL related to HAE. While this could have potentially resulted in a selection bias and limited our understanding of how participants might compare to other adolescents with HAE, especially in terms of disease severity and HRQoL impacts, other steps were implemented (requirement of participants to be taking prophylaxis and to provide confirmation of diagnosis) to recruit and enroll a representative sample. This study also had a number of strengths. First, the sample was composed of a balanced representation of age groups and self-identified sex at birth. Next, this study used one-to-one interviews, which aligns with recommendations for conducting qualitative interviews with adolescent populations as it encourages participants to share their personal experiences and limits the likelihood of social desirability bias [25, 26]. Finally, this study is among the first to use qualitative research methods to present an in-depth description of the adolescent experience of HAE.

## Conclusions

Adolescents with HAE reported experiencing a range of symptoms that, when untreated, impacted their overall HRQoL. While similar findings have been reported in adult populations, those described here indicate that certain impacts are specific to adolescents, in particular those related to social and emotional functioning and schooling. Further, adolescents reported that effective treatments (prophylactic and acute) inhibited symptoms and HRQoL impacts with minimal treatment burden. These learnings suggest that health care providers and clinical investigators should consider the unique HRQoL impacts experienced by adolescents when evaluating treatment benefit.

## Data Availability

The raw data generated and/or analyzed during the current study (i.e. interview transcripts) are not publicly available due to confidentiality and consent limitations. The interview guide can be made available upon request.

## Footnotes

1 This requirement was included to help prevent enrollment of patients who were not taking prophylaxis because they experienced more mild symptoms and related impacts on HRQoL.

## Abbreviations

AE: Angioedema
HAE: Hereditary angioedema
HRQoL: Health-related quality of life
PAG: Patient advocacy group
U.S.: United States
